# Acute dorsal myelopathy resulting from intramedullary cysticercus: a case report

**DOI:** 10.1186/s13256-021-02693-w

**Published:** 2021-03-17

**Authors:** Durjoy Lahiri, Abhishek Chowdhury, Souvik Dubey, Biman Kanti Ray

**Affiliations:** Department of Neurology, Bangur Institute of Neurosciences, IPGMER and SSKM Hospital, Kolkata, 700025 India

**Keywords:** Cysticercosis, Disseminated, Intramedullary, Acute myelopathy

## Abstract

**Background:**

Neurocysticercosis is the most common parasitic infection of the central nervous system, brain being the most frequent site. Intramedullary location of cysticercus is a rarely described entity in literature. Widespread dissemination of cysticercus is also considered a rare occurrence, and only a handful of cases are documented, almost exclusively from tropical nations. Here we present a case of disseminated cysticercosis with rare initial presentation as acute dorsal myelopathy resulting from intramedullary cysticercus.

**Case presentation:**

A 62-year-old male patient from India (Asian) presented with features of dorsal myelopathy as manifested by acute-onset symmetric paraparesis, sensory loss below umbilicus, and double sphincter dysfunction. General physical examination revealed pea-sized nodules in skin and tongue. On spinal cord imaging, it was found that he had intramedullary cysticercus with diffuse perilesional edema. Brain and muscle imaging showed extensive cysticercosis suggestive of dissemination. Histological examination from skin nodule and antibody assay confirmed the diagnosis of cysticercosis. Following steroid administration, our patient showed improvement with observable increase in power of the lower limbs. He was subsequently discharged with antiepileptics, symptomatic therapy, and regular physiotherapy. Antihelminthic agents were initially avoided in view of extensive cysticercosis in brain including crucial areas such as brainstem.

**Conclusion:**

Rare manifestation of a rare but treatable disorder makes it an important reportable observation in the context of tropical medicine.

## Background

Cysticercosis is caused by *Cysticercus cellulose*, a larval form of pork tape worm *Taenia solium*. Humans are the only definitive hosts, and pigs are the usual intermediate hosts. Humans acquire infection following ingestion of *T. solium* eggs, usually from a tapeworm carrier [1]. Clinical presentation depends on the number and location of cysticerci and associated inflammatory responses as well as scarring. Brain parenchyma, Cerebrospinal fluid (CSF), skeletal muscles, subcutaneous tissue, and eye are considered among the common sites for cysticerci [2]. Brain is frequently vulnerable to cysticercosis to the extent that cysticercus is considered the most common parasitic infection affecting brain [3]. Owing to its multifocal nature of involvement in brain parenchyma, relative absence of focal neurological signs is a typical feature in neurocysticercosis (NCC).

Disseminated cysticercosis (DCC) is a rare entity. Dissemination of cysticerci throughout the human body was reported as early as 1912 by British Army medical officers posted in India [4]. Since then, however, very few cases of DCC have been documented in literature, and the tally so far remains around 50 cases worldwide [5]. A study from a government medical college in northern India reported only one case of DCC among their collection of 450 cases of cysticercosis [6]. Usual presentations include seizures, hydrocephalus, dementia, muscle enlargement, and subcutaneous and lingual nodules.

Acute dorsal myelopathy is a commonly encountered neurological emergency. Demyelination and vascular pathologies are usually given priority in the list of differentials. Infective causes of acute dorsal myelopathy, however, are also not infrequent, particularly in tropical countries. Intramedullary cysticercus, a rare entity reported almost exclusively from tropical regions, usually presents as chronic progressive myelopathy [7–9]. Interestingly, none of the so far reported cases has described this entity as part or presentation of disseminated cysticercosis, which may be linked to actual rarity of both the conditions.

Here we describe a case of DCC with rare initial presentation as acute dorsal myelopathy resulting from intramedullary cysticercus. Rare manifestation of a rare but treatable disorder makes it an important reportable observation in the context of tropical medicine.

## Case presentation

**Fig. 1:**
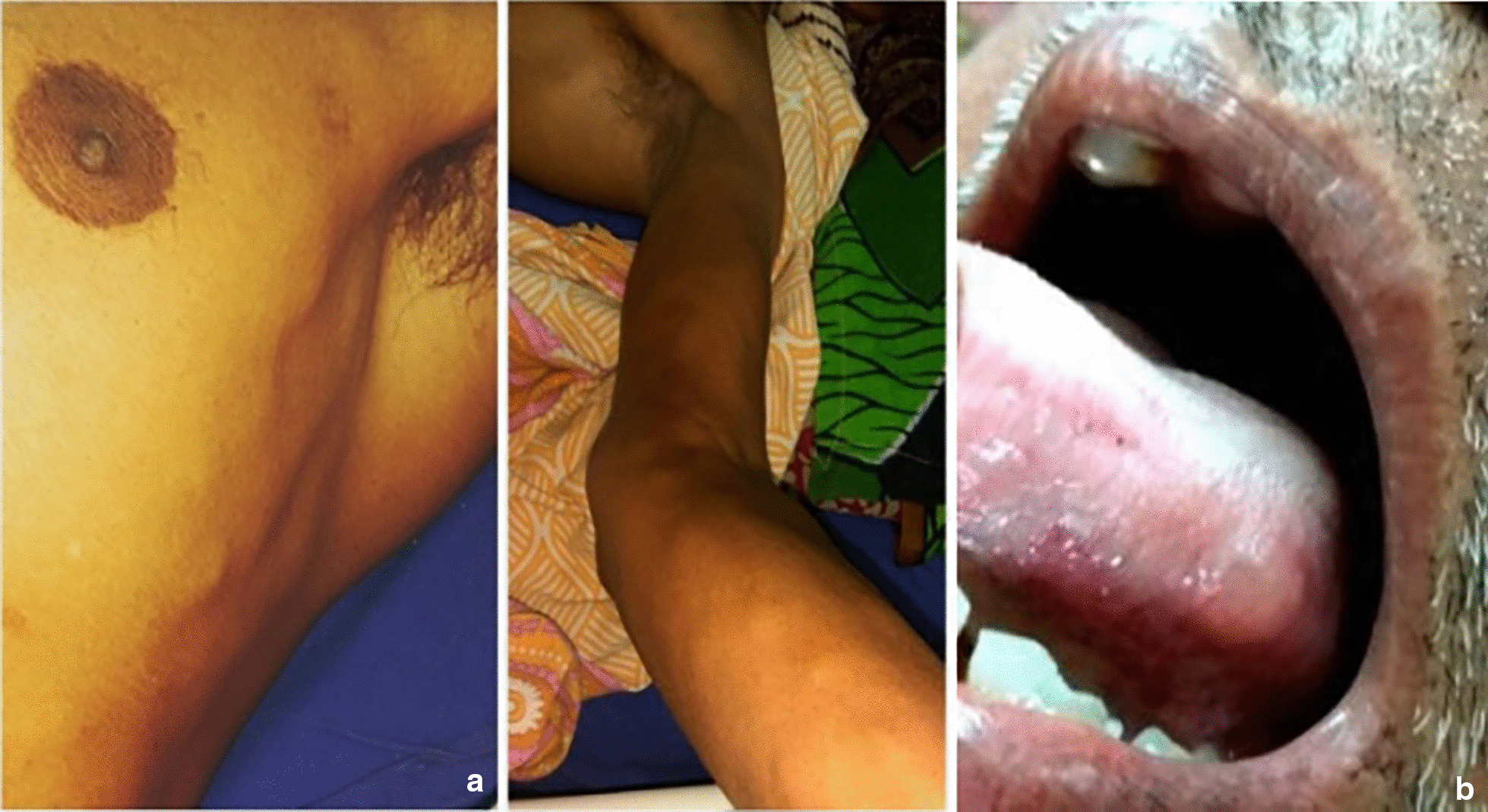
**a** Multiple pea-sized nodules over arm and chest. **b** Nodule over left lateral border of tongue

A 62-year-old male patient from rural Bengal (India), farmer by occupation, presented with acute-onset nonradiating low-back pain followed by weakness and numbness of both lower limbs for 1 month. There was associated double sphincter dysfunction, for the same duration, in the form of acute painless retention of urine and constipation. He was put on a catheter on the third day of illness that remained *in situ* at the time of admission to our care. On detailed questioning, he revealed that lower limb weakness started from left side in a hololimb manner with rapid involvement of right lower limb within subsequent 24 hours. His sensory symptom, which was in the form of loss of sensation in both lower limbs and lower trunk up to umbilicus, started at around the same time, although in a more symmetrical manner. At the time of presentation, he specifically complained of a girdle-like sensation around the umbilicus, below which he felt diminished sensation to touch or any other stimulus. All his complaints remained approximately static since the onset of illness. There was no history suggestive of any cranial nerve involvement, seizures, headache, vomiting, or loss of consciousness. There was no history of associated fever, cough, hemoptysis, weight loss, trauma, or dimness of vision. He used to eat cooked mutton, fish, chicken, and raw vegetables. General examination revealed multiple pea-sized nodules all over the body, especially both forearms, arms, and chest (Fig. 1a) and one on left lateral border of tongue (Fig. 1b). His pulse was 80/minute, regular, and blood pressure was 110/80. Neurological examination revealed a Glasgow Coma Score of E4V5M6, no meningeal signs, and normal cranial nerve examination. Fundoscopy also was unremarkable. Motor examination revealed normal bulk, with decreased tone in bilateral lower limbs and normal tone in both upper limbs. Power was 0/5, and deep tendon reflexes were absent in bilateral lower limbs with normal upper limbs. There was loss of all sensation in his lower limbs, with a sensory level corresponding to T9 dermatome, in addition to impaired joint position sense. There was no detectable gibbus or any spine tenderness.

**Fig. 2:**
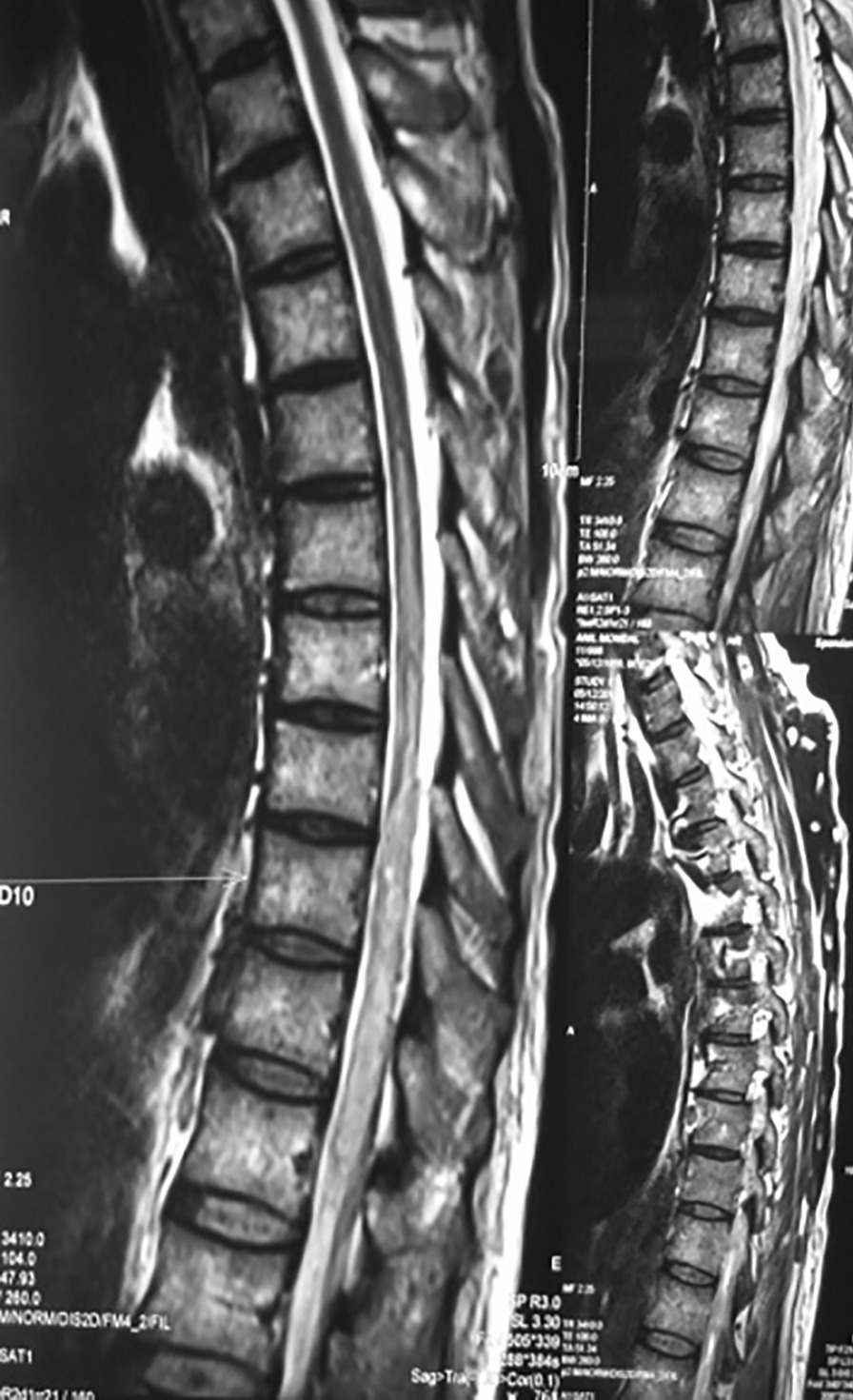
Magnetic Resonance Imaging of dorsal spine (plain and contrast) showing diffuse cord swelling with edema in the lower dorsal spinal cord extending up to conus medullaris accompanied by patchy enhancement within. Extensive neurocysticercosis within both paravertebral as well as posterior paraspinal muscles is visible. (Arrow shows the position of 10th thoracic vertebra)

**Fig. 3:**
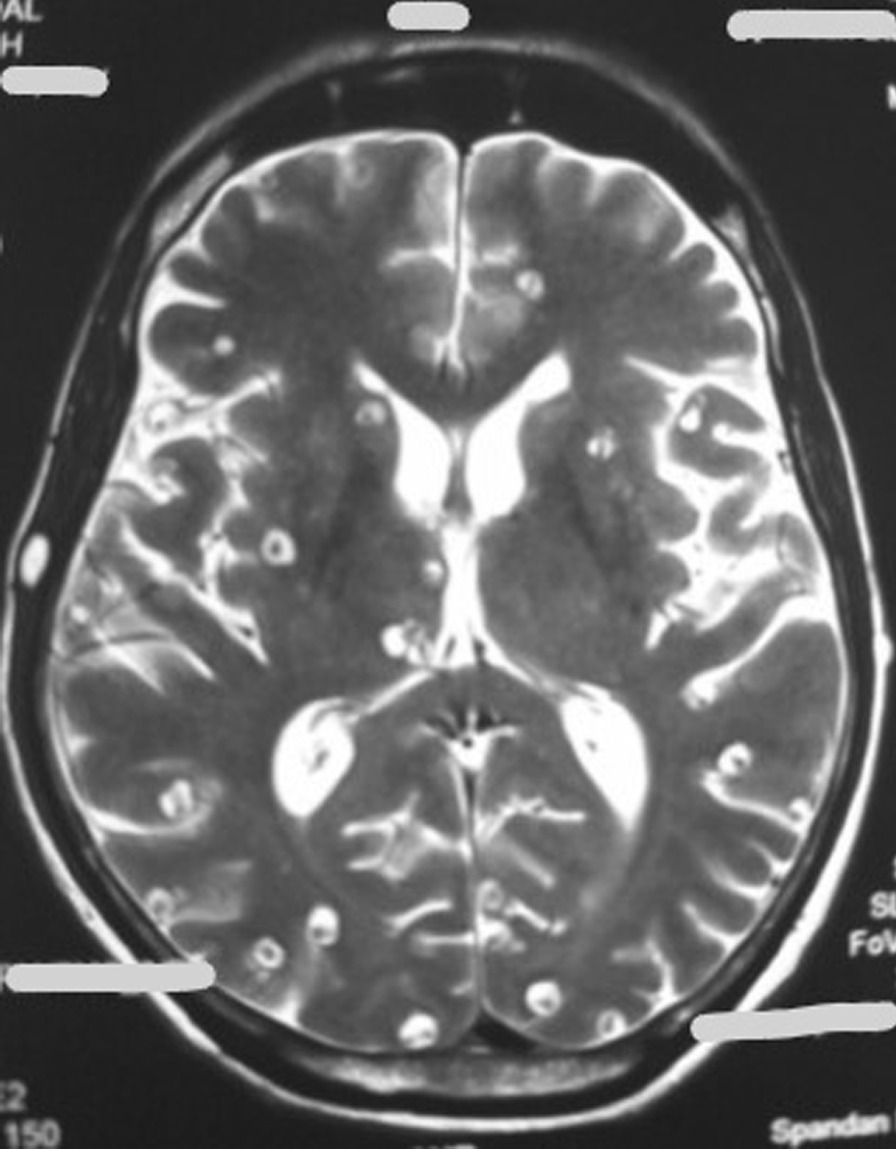
Magnetic Resonance Imaging of brain (T2 sequence) showing multiple cysts with scolex contained therein

**Fig. 4:**
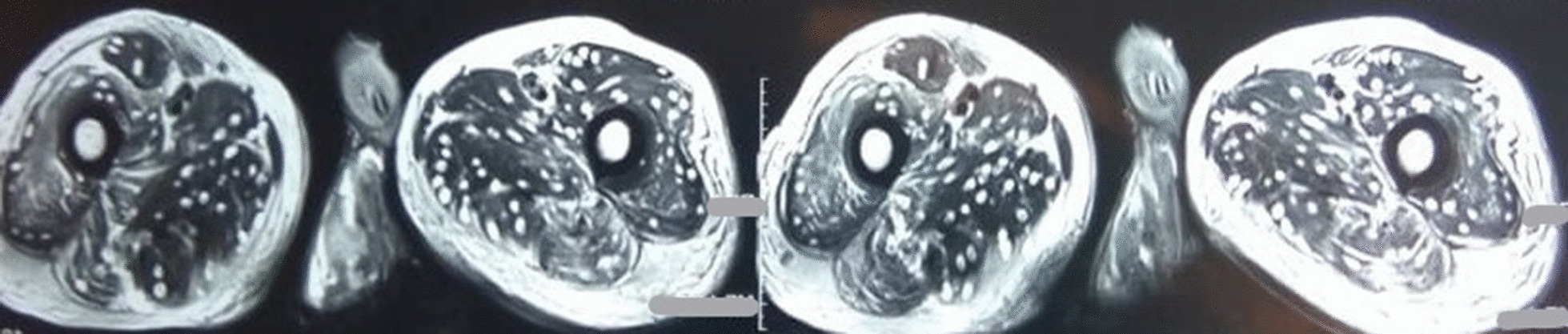
Magnetic Resonance Imaging of thigh muscles showing numerous cysticerci

**Fig. 5:**
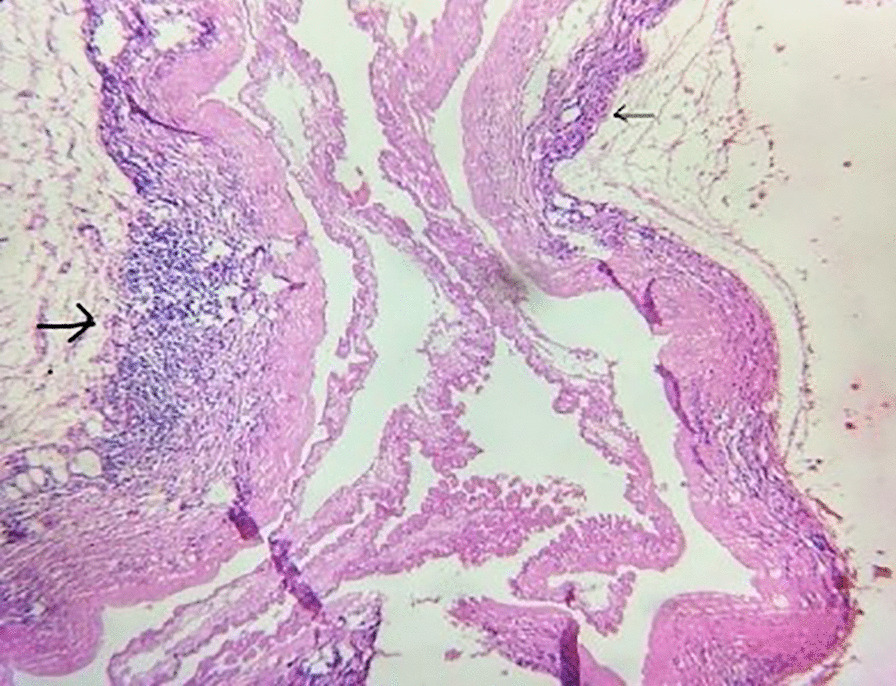
Histopathological examination from skin nodule biopsy showing numerous cysts (black arrows) in the subcutaneous layer

Routine investigation revealed hemoglobin 11.4 gm%, total leukocyte count 7100, differential count N_61_L_24_E_12,_ absolute eosinophil count 852, and platelet of 1.75 lakhs. Liver and renal function tests were within normal limits. Fasting blood sugar was 86 mg/dl. Magnetic resonance imaging (MRI) of dorso-lumbar spine with contrast, which was done outside approximately 1 week following symptom onset, showed small rim enhancing lesion in the spinal cord at D11 level with edema in the surrounding cord as well as diffuse cystic lesions in skeletal muscles and brain parenchyma consistent with cysticercosis. On admission, a repeat MRI of dorsal spine (plain and contrast) showed diffuse cord swelling with edema in the lower dorsal spinal cord extending up to conus medullaris and showing patchy enhancement within. It also showed extensive neurocysticercosis within both paravertebral as well as posterior paraspinal muscles (Fig. 2). Report of cerebrospinal fluid (CSF) examination was as following: protein 129 mg/dl, glucose 75 mg/dl, cell count 30 cells/ml (98% mononuclear), and few erythrocytes. CSF adenosine deaminase was within normal limits, and culture did not reveal any growth. Patient was immediately put on intravenous injection methylprednisolone 1 g/daily for 5 consecutive days followed by oral steroids. Meanwhile, MRI of brain (plain + contrast) revealed multiple elliptical rim-enhancing lesions with few showing eccentric internal dot within both cerebral hemispheres, both cerebellar lobes, and within orbits, suggestive of neural and ocular cysticercosis (Fig. 3). MRI of both thighs showed numerous elliptical rim-enhancing lesions with few showing eccentric internal dot within bilateral thigh region, giving a starry-sky appearance (Fig. 4). Histological examination of a nodule excised from the right arm showed numerous cysticerci in the subcutaneous tissue (Fig. 5). The immunological enzyme-linked immunosorbent assay test for cysticercal antibodies was positive, and a diagnosis of DCC was reached in this case.

Following intravenous steroid administration, within 5–7 days our patient showed improvement with observable increase in power of the lower limbs up to the extent of 3/5. He was subsequently discharged with antiepileptics (tablet levetiracetam 500 mg twice daily), symptomatic therapy, and regular physiotherapy. Antihelminthic agents were avoided in view of extensive cysticercosis in brain including crucial areas such as brainstem. The patient was unfortunately lost in follow up after 3 months following discharge from our care.

## Discussion

NCC is the most common parasitic disease of the human central nervous system. Cysticercal spinal cord involvement, however, is infrequent and varies from 1–5 % of all cases of neurocysticercosis [7]. Spinal involvement can be in vertebral, extradural, intradural, or intramedullary form. The usual way in which NCC involves the spinal cord is from an extramedullary (racemose) formation, as a result of dissemination of the vesicles through the subarachnoid space from the posterior cranial fossa [8]. Intramedullary involvement is extremely rare, with only a handful of reported cases. Intramedullary extension of cysticercosis probably takes place through arterial circulation and is directly proportional to regional blood flow. Likewise, the most common region of intramedullary cysticercosis is thoracic, followed by cervical, lumbar, and sacral regions [9]. Neurological manifestation of spinal cord involvement in NCC can be due to either irritation or mass effect. Typical presentation of spinal cord neurocysticercosis is in the form of compressive myelopathy with a chronic progressive clinical course.

To the best of our knowledge, the presented case is the first report of intramedullary neurocysticercosis that manifested as acute dorsal myelopathy mimicking transverse myelitis. The clinical journey of our patient can be interpreted as follows: intramedullary compressive dorsal myelopathy resulting from cysticercus and manifesting as transverse myelitis followed by possible irritation of surrounding cord tissue or cyst rupture giving way to extensive perilesional edema that might have contributed to the static course of neurological deficits in this case. The observable clinical improvement following steroid introduction might have been linked to partial subsidence of edema.

Another curious aspect of this case is eventual detection of DCC, which in itself is an uncommon occurrence even in tropical regions. Among the few reported cases of DCC, the majority have been from India [5]. The organs most frequently affected are subcutaneous tissue, skeletal muscles, lungs, brain, eye, and liver. Heart, thyroid, and pancreas are occasionally reported to be involved in the disease process. For reasons that are not obvious, spinal cord involvement is considered rare from the perspective of DCC. In fact, intramedullary cysticercus as the initial presentation of disseminated disease has not been reported previously in literature.

The treatment of DCC consists primarily of symptomatic therapy accompanied by steroids and antiepileptic drugs. Antihelminthic drugs help to reduce parasite burden faster, although death of parasites may eventually occur without pharmacological intervention as well [3]. Praziquantel (10–15 mg/kg/day for 6–21 days) and albendazole (15 mg/kg/day for 30 days) are the recommended cysticidal drugs. Of note, parasite death with subsequent antigen release may trigger allergic reactions that can potentially lead to deterioration of clinical status, particularly if cysts are located in crucial areas such as spinal cord, brainstem, or eye. Thus, introduction of cysticidal agents in DCC usually requires caution on part of the treating physician. In the present case, pharmacological killing of parasites was avoided in view of intramedullary location of the cyst, which had already once manifested as transverse myelitis.

## Conclusion

To the best of our knowledge, this is the first reported case of DCC that presented with acute dorsal myelopathy consequent to intramedullary cysticercus. Although intramedullary cysticercosis itself has been reported earlier in literature, the combination of DCC and acute dorsal myelopathy makes this case unique. Furthermore, the essence of this case lies not only in the fact that it represents a rare manifestation of a rare disease, namely DCC, but also in the certainty of its treatable nature.

## Data Availability

Data sharing not applicable to this article as no datasets were generated or analyzed during the current study.

## Abbreviations

DCC: Disseminated cysticercosis
NCC: Neurocysticercosis
MRI: Magnetic resonance imaging
CSF: Cerebrospinal fluid
